# Health literacy and self-care among adult immigrants with type 2 diabetes: a scoping review

**DOI:** 10.1186/s12889-024-20749-6

**Published:** 2024-11-22

**Authors:** Christine Tørris, Line Nortvedt

**Affiliations:** Department of Nursing and Health Promotion, Faculty of Health Sciences, Oslo Metropolitan University, Oslo, 0130 Norway

**Keywords:** Health literacy, Self-care, Self-management, Quality of life, QoL, Adult, Diabetes, Cardiovascular risk

## Abstract

**Introduction:**

There exists a gap in the health status of immigrants in comparison to the overall population, and health literacy has been shown to be a mediator for health outcomes and may predict their quality of life (QoL). We aimed to systematically map and synthesize research findings on adult immigrants’ health literacy in terms of their health beliefs, understanding, and self-management of Type 2 Diabetes Mellitus.

**Methods:**

A scoping review guided by Arksey and O’Malley’s framework was conducted, based on systematic searches in the Embase, Ovid MEDLINE, and APA PsycInfo databases in June 2023. The retrieved articles were screened and assessed by the two authors independently.

**Results:**

Of 568 identified studies, 16 (9 qualitative, 4 cross-sectional, 1 mixed-methods, and 2 experimental) were included in this review. Low/moderate health literacy levels with no sex-related differences were reported. Immigrants’ access to health information was limited by language barriers and a lack of culturally adapted information, especially from their physicians. Among women, access to health information was limited by patriarchal norms. Knowledge gaps were primarily related to understanding the necessity of medication and the importance of a healthy lifestyle. Healthcare professionals played an important role in motivating immigrants to adhere to treatment.

**Conclusion:**

Few studies were found on this topic, and additional research is needed to enhance health literacy among immigrants. Limited health information, language barriers, and a shortage of culturally sensitive knowledge appear to hinder immigrants’ ability to access, understand, and apply health information. Cultural norms and personal factors further suppress these abilities, ultimately impacting their health outcomes. The findings of this study suggest that health literacy is a crucial component of healthcare professionals’ curricula, equipping them with the skills to identify and assist patients with low health literacy.

## Introduction

Non-communicable diseases, such as cardiovascular diseases (CVDs), cancer, and diabetes, continue to pose major challenges [1]. Approximately 537 million adults worldwide have diabetes, with Type 2 diabetes (T2DM) accounting for nearly 90% of cases [2, 3]. T2DM is associated with obesity, where rapid economic development has led to urbanization and a more sedentary lifestyle together with unhealthy eating patterns [4]. The accumulation of excessive body fat due to a positive energy balance leads to metabolic and homoeostatic disturbances associated with the pathogenesis of T2DM, such as organ dysfunction and premature death due to microvascular (e.g., retinopathy, neuropathy, and nephropathy) and macrovascular complications (e.g., ischemic heart disease, cerebrovascular disease, and peripheral vascular disease) [2, 3, 5]. Such complications often occur alongside increased glycemic variability and suboptimal glycemic control. Therefore, early detection and proactive management of T2DM (e.g., glycemic and obesity management) are essential to improve health outcomes [2].

A higher prevalence of T2DM has been seen in some ethnic groups (e.g., African American, Latino, American Indian, Asian, African, and Pacific Islander) [2], and T2DM has become a great challenge for immigrants and refugees (hereafter called immigrants) [1, 6, 7]. In addition to genetic predisposition, several interacting causes including changing from a poor to an affluent environment, increase the prevalence of T2DM among immigrants [6]. For example, a 55% higher risk of having diabetes has been observed in immigrants than in locals of similar age, sex, and place of residence in Italy [8]. In Great Britain, the prevalence of T2DM among migrants of South Asian origin has been reported to be around 20%, which is nearly five times higher than that of the locale population in Europe [9]. Moreover, a recent review found a prevalence of 12% among first-generation Chinese immigrants [10]. According to an integrative review of health status in the Nordic countries, older immigrant women may have worse health indicators than their male counterparts [11]. A Danish cohort study found that immigrants were more affected by diabetes and had a higher risk of comorbidities than locale individuals [12]. A Canadian study reported similar findings [13]. A Norwegian register-based study found that the risk of multimorbidity doubled among immigrants after a five-year stay in the country, with a higher prevalence of comorbidities among women [14].

A disparity exists in the health status of immigrants compared to the overall population [15], and health literacy (HL) has been identified as a mediating factor for health outcome [16] and may also predict diabetes patients’ quality of life (QoL) [17]. Specifically, low HL has been associated with a higher risk of T2DM [18], as well as impaired ability to achieve glycemic control. Therefore, self-care interventions aimed at improving HL could be crucial for supporting health-related decision-making, advancing patients’ right to health and enhancing their well-being [19].

HL refers to the capacity to meet health needs and is defined as the “ability of an individual to obtain and translate knowledge and information to maintain and improve health in a way that is appropriate to the individual and system contexts” [20]. Nutbeam extends this concept to encompass personal, cognitive, and social skills that determine an individual’s ability to utilize health information for health promotion and maintenance [21, 22]. Here, health promotion outcomes represent personal, social, and structural factors that can be modified to change the determinants of health (intermediate health outcomes such as lifestyle, access to health services, and a healthy environment) [22]. Sørensens’ integrated HL model (2012) outlines the competencies related to accessing (seeking, finding, and obtaining), understanding (comprehending), appraising (interpreting, filtering, judging, and evaluating), and applying (communicating and using) health-related information [23]. HL may positively influence health behaviors by reducing perceived barriers promoting a healthy lifestyle and better decision-making [24]. Even though HL can’t singlehandedly solve health disparities, it can help mitigate these causes and empower individuals to better manage their health determinants [16].

Future interventions should particularly focus on developing generic, transferable skills, positioning HL as both a personal and societal resource [16]. This person-centered approach includes components such as cultural appropriateness and skill building, empowering individuals to manage their health and factors influencing health-related outcomes [25]. Here, targeting high-risk groups is vital but insufficient to address deep-rooted disparities in power, resources, and opportunities [25], and systematic responses that enhance HL variably across social levels are needed [16]. This concept, known as ‘proportionate universalism,’ advocates for support tailored to the degree of need, suggesting that health interventions should be universal, not selectively targeted, and implemented with a scale and intensity that is proportionate to the level of disadvantage experienced by individuals [26].

Health professionals provide equal services to patients, taking into account patient-centered factors such as diabetes knowledge and self-management (e.g., medication adherence and glucose checks) [27]. In this context, health professionals can enhance patients’ HL by making health information about their conditions more accessible, understandable, and applicable [28]. However, little is known about immigrants’ HL and their self-management of T2DM.

This study aims to systematically map and synthesize research findings on adult immigrants’ HL regarding their health beliefs, understanding, and self-management of T2DM.

## Methods

### Study design

This scoping review was guided by Arksey and O’Malley (2005) [29] and Levac (2010) [30]. The work proceeded in six stages: 1: formulating a research question, 2: identifying relevant studies, 3: selecting data, 4: charting the data, 5: collating, summarizing, and reporting the results, and 6: consulting with stakeholders. The Preferred Reporting Items for Systematic Reviews and Meta-Analyses Extension for Scoping Reviews (PRISMA-ScR) [31] and the methodology described by the Joanna Briggs Institute [32] were used to report the results. The protocol is available from the corresponding authors upon reasonable request.

### Stage 1: Research questions

This review addressed the following research question: *What is known about adult immigrants’ HL related to their self-management of T2DM?*

A particular focus was on immigrants’ countries of origin and places of residence, possible sex differences, barriers to and facilitators of HL, knowledge levels, and health beliefs. The research question was guided by the population, intervention, comparison, and outcome (PICO), and population, concept, and context (PCC) frameworks [31, 32].

### Stage 2: Identifying relevant literature

**Fig. 1:**
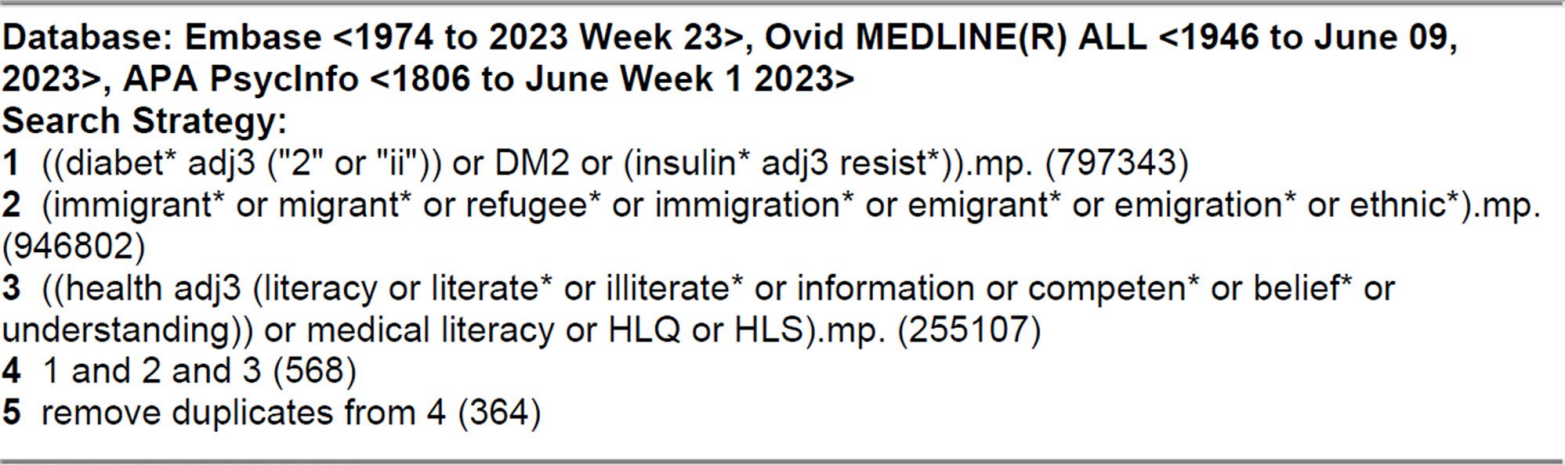
Search terms

Searches were performed in Embase, Ovid MEDLINE, and APA PsycINFO databases in June 2023. The searches were restricted to papers written in English or Scandinavian (i.e., Norwegian, Swedish, and Danish) languages (Fig. 1). The search process was planned in collaboration with a librarian, engaged to help define the parameters of the search strategy, and undertake the searches of the relevant databases.

### Stage 3: Study selection

The study inclusion and exclusion criteria (Table 1**)** were based on the specific research question, and guided by PICO and PCC [32]. Screening and reviewing were performed by both authors so that all papers would be checked by two reviewers. Each reviewer independently screened candidate articles for eligibility. Disagreements were resolved through discussion between the two authors. If no agreement could be reached, a third researcher would decide, but this proved unnecessary.

After duplicates were removed, the studies from the search results were downloaded to EndNote and imported into Covidence literature screening software to achieve a streamlined review process in all study phases. The article titles and abstracts were screened for eligibility by the two authors independently followed by the full-text assessment.

**Table 1:** Study inclusion and exclusion criteria

PICO	Inclusion criteria	Exclusion criteria
Participants	≥ 18 years of age Immigrants or refugees T2DM or insulin resistance	< 18 years of age Not immigrants/refugees Conditions other than T2DM
Intervention	Not applicable	
Comparison	Not applicable	
Outcome/Concept	HL (health information, health competence, health beliefs, health understanding), medical literacy, HLQ or HLS	
Context	Healthcare setting	
Study design	Qualitative, quantitative, and mixed-methods	Conference paper abstracts, dissertations, protocols, reviews, case studies
Language	English and Scandinavian languages*	

HL: Health literacy, HLQ: Health literacy questionnaire, HLS: Health literacy survey, T2DM: Type 2 diabetes

*Scandinavian languages: Norwegian, Swedish, and Danish

### Stage 4: Charting the data

After completing the screening process, key information on the included studies was extracted. This included *the first author’s name*,* year of publication*,* origin*,* aim*,* data collection methods*,* participants*, and *main findings*. Relevant information according to the research question related to *HL* and immigrants’ *access to*, *understanding of*, and *use/application* of health information to promote good health was collected and organized to enable analysis and synthesis [22]. Quality assessment is not within the scope of the scoping study remit and was therefore not conducted in this review, consistent with the proposed scoping review methodology [31, 32].

### Stage 5: Collating, summarizing, and reporting the results

This stage was conducted in three steps as recommended by Levac [30]: (a) descriptive numerical summary and qualitative deductive content analysis, (b) reporting results referring to the research question, and (c) the findings related to the overall study purpose and broader implications for future practice [30]. The included articles were analyzed using the theoretical model of Sørensen (2012), where the competencies implied by HL (accessing, understanding, and use/applying of health information) are used to structure the coding process [23].

### Step 6: Consultation with stakeholders

To stimulate discussion and receive feedback on our process and preliminary findings, we engaged a professor specializing in migration health to provide ongoing feedback throughout the study.

## Results

### Selection of studies

**Fig. 2:**
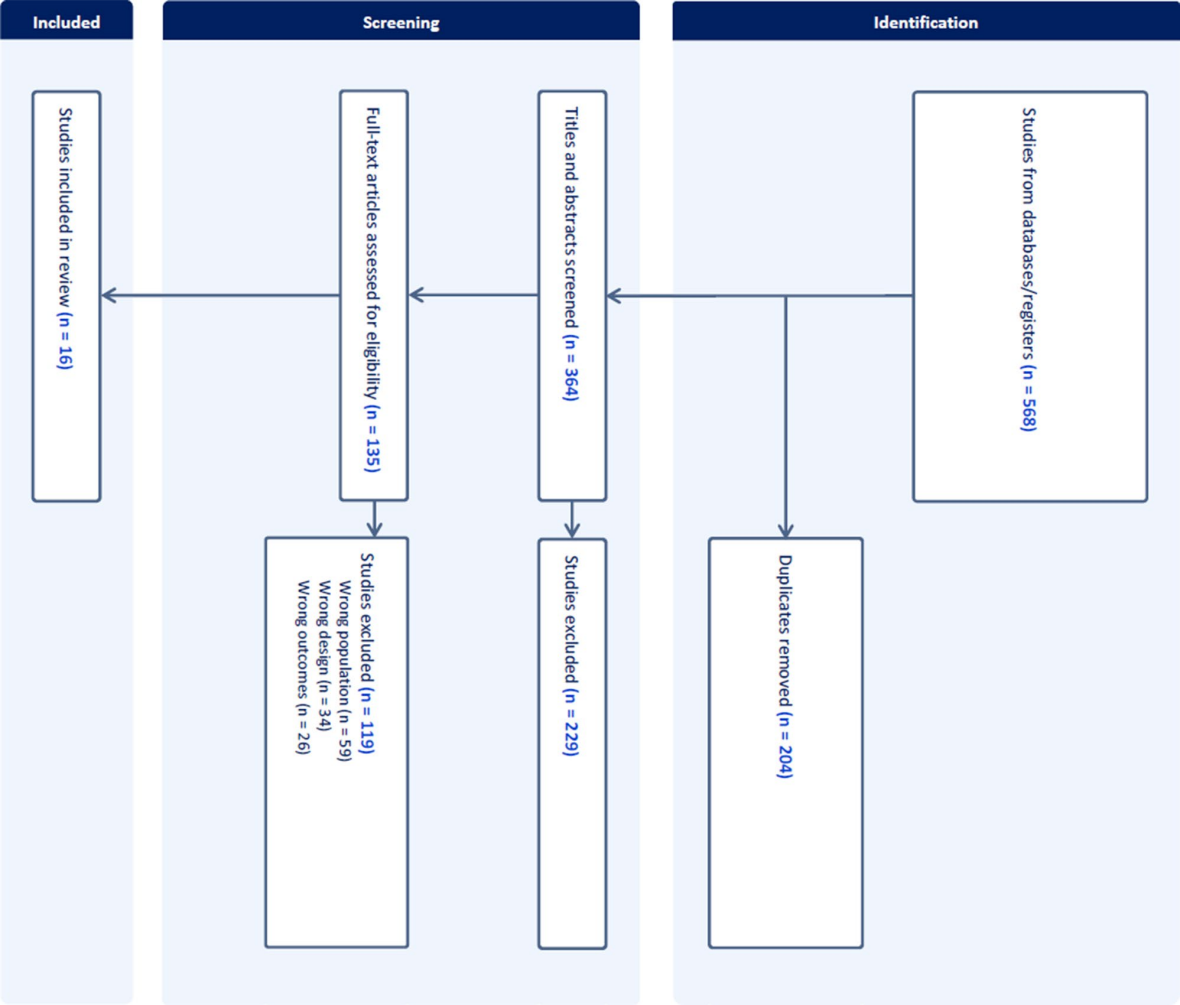
PRISMA flowchart

The electronic database searches yielded 568 candidate studies. After duplicates were removed, 364 studies remained. Based on their titles and abstracts, 229 were excluded. Thus, 135 full-text articles were retrieved and assessed for eligibility. Of those, 119 were excluded, mostly due to the wrong population. Thus, 16 studies were included in this scoping review (Fig. 2).

### Characteristics of the included studies

All included articles were in English, and published between 2001 and 2023 [33–48]. Nine of the reviewed studies were qualitative [34, 35, 37–39, 41, 43, 45, 48], four cross-sectional [33, 42, 44, 47], one had a mixed-methods design [46], and two experimental [36, 40]. Smith-Miller et al. conducted multiple studies using the same dataset [44–46] (Table 2).

**Table 2:** Characteristics of the included studies (*n* = 16)

First author, year Country	Aim	Data collection	Participants*	Main findings	Findings related to HL and aspects of HL: access (A), understanding (U), and use/apply (UA)			
**Qualitative**					**HL**	**A**	**U**	**UA**
Ahmad, 2021 [34] Australia	To explore medication-taking behaviors and factors influencing the phases of adherence (initiation, implementation, discontinuation)	Semi-structured interviews	Convenience sample of Indian migrants in Sydney (*n* = 23, 5 women, 33–72 years old)	Factors influencing adherence: 1) Initiation: Most patients started taking medicine while some delayed it due to fear of side effects and medication dependency 2) Implementation: Most patients reported taking medicine as prescribed, while some reported forgetting their medication 3) Discontinuation: Some patients discontinued their medication in favor of Ayurvedic medicine			X	X
Biyikli Gültekin, 2017 [35] Austria	To explore difficulties related to the diagnosis, treatment, and experiences of T2DM (needs, expectations, special conditions, and cultural characteristics)	Semi-structured interviews	Turkish immigrants were recruited from Turkish mosques in Vienna (*n* = 13, all women, 37–61 years old)	A lack of knowledge delayed diagnosis and affected the approach to T2DM management (i.e., culturally the meal is a social arena). Due to language barriers, the most common sources of health information were Turkish television, newspapers, magazines, friends working in the healthcare sector, and patient training sessions with Turkish physicians		X	X	
Cokluk, 2023 [37] Norway	To describe T2DM self-management experiences of Turkish immigrants in Norway	Three focus group interviews	Turkish immigrants (*n* = 13, 9 women, 41–71 years old)	Three major themes: 1) Understanding the responsibility and role of healthcare staff in treating T2DM 2) Assessing T2DM education courses and information provided in the courses 3) Applying knowledge and motivation to adapt to life with T2DM Self-management was related to cultural, religious, and socioeconomic backgrounds		X		X
Jamil, 2022 [9] USA	To explore the impact of cultural perspectives on medication adherence for T2DM and CVD	Individual semi-structured interviews DOSE-Nonadherence measure (1–5, 1: adherent)	South Asian immigrants (from Pakistan, India, and Bangladesh) (*n* = 12, 6 women, 49–75 years old)	Medication adherence was good (mean 1.36) Five themes: 1) Clear numerical goals for their blood-sugar motivated health-related changes 2) Open communication improved medication adherence 3) Self-management and autonomy were valued 4) Religious/spiritual beliefs might promote medication adherence 5) Complementary/alternative medicines augmented Western medicines			X	
Jayne, 2001 [39] USA	To demonstrate the application of a Self-Regulation Model. To identify beliefs about health and illness	Individual interviews	Chinese immigrants recruited from a West Coast Chinatown health center (*n* = 30, 13 women, 46–80 years old)	The participants did not have a clear picture of the etiology and chronicity of diabetes and perceived the illness as stigmatizing Coping strategies included wishful thinking, belief in powerful others, keeping diabetes a secret, and avoiding social situations. Participants could not appraise their coping strategies			X	
Leung, 2014 [41] USA	To explore immigrants’ difficulties in obtaining, processing, and understanding diabetes-related information despite translations	Six focus groups and two individual semi-structured interviews	Chinese immigrants recruited from community health centers, in Los Angeles, California. (*n* = 29, 11 women, ≥ 45 years old)	Cultural (high regard for authority, avoiding being burdensome, desire to follow a collective approach), structural (insurance, transportation, limited information in the community), and personal barriers (unawareness of self-care responsibility and age-related limitations) affected the capacity to obtain, communicate, process, and understand diabetes-related health information which influenced decision-making and self-care		X		
Sharma, 2022 [43] USA	To explore the impact of patriarchy on women’s diabetes self-management	Semi-structured interviews (phone)	Bhutanese immigrants recruited from a Medical Center (*n* = 15, all women 20–75 years old)	Family structure, religious beliefs, traditional healthcare, and gender roles determined women’s patriarchal upbringing and lifestyle. Immigration led to poor socioeconomic indicators and marginalization, and women’s access to healthcare relied on family members Women experienced side effects and their ability to maintain a healthy lifestyle was determined by physical health, knowledge of dietary practices, and self-efficacy		X	X	
Smith-Miller 2017 [45] USA	To investigate the understanding of T2DM self-management	Individual semi-structured interviews	Latino immigrants recruited through flyers (*n* = 30, 19 women, 27–86 years old)*	The participants considered T2DM a serious disease. The importance of exercise and diet was well understood, while medication adherence was not. Negative interactions/communication with healthcare providers, cultural stigma related to the disease, financial constraints, immigrant status, and the complexity of the disease were barriers to self-management		X	X	
Washington, 2009 [48] USA	To explore self-care practices and risk factors related to lifestyles, attitudes, and health beliefs	Individual in-depth semi-structured interviews	Chinese immigrants recruited through flyers (*n* = 13, 6 women, ≥ 65 years old)	Health beliefs, attitudes, and diabetes self-care management were influenced by healthcare practitioners’ ability to provide culturally sensitive resources			X	
**Cross-sectional**					**HL**	**A**	**U**	**UA**
Abuelmagd, 2018 [33] Norway	To assess how Pakistani women in Norway lived with T2DM and how they responded to lifestyle and medical information	Individual structured (questionnaire-based) interviews	First-generation Pakistani immigrants recruited through key representatives of Pakistani women groups, nursing homes, and mosques (*n* = 120, all women, 29–80 years old)	The participants reported suboptimal diabetes control (lifestyle habits, comorbidities, and drug use). Most (75%) reported requiring assistance to understand written medical information; 27% were illiterate and reported poor health; 71% had macrovascular comorbidities, 24% altered drug intake due to religious fasting; 32% were unable to measure their blood glucose levels themselves; and 25% used insulin in addition to oral medication		X	X	
Njeru, 2016 [42] USA	To assess diabetes-related HL, and associations with diabetes outcomes	SKILLD scale to measure HL (0–100), low HL score < 50	Somali immigrants/refugees recruited during scheduled clinical encounters (*n* = 50, 62% women, mean age 52.5 years SD 16.01)	Low diabetes-related HL (mean SKILLD score 42) was observed and associated with higher age, interpreter needs, lower income, and diabetes-related complications. No associations between HL scores and diabetes outcome measures: HbA1C, LDL cholesterol, and blood pressure were found	X			
Smith-Miller 2016 [44] USA	To assess HL, diabetes knowledge, and the factors affecting T2DM self-management	SAHLSA to measure HL (0–50, ≤ 37 = low HL), and DKT (0–24) to measure diabetes knowledge	Latino immigrants recruited through flyers (*n* = 30, 19 women, 27–86 years old)*	Moderate HL score: SAHLSA mean (SD) 38.4 (8.5), and low DKT mean (SD) 12.0 (2.5) were observed. Knowledge and healthy lifestyle behaviors (health responsibility, physical activity, nutrition, spiritual growth, and interpersonal growth) were important components of T2DM self-management	X			
Tatara, 2019 [47] Norway	To explore the use of eHealth for T2DM self-care management and related user factors (language, length of residence, and diagnosis)		Pakistani immigrants recruited through purposive sampling (*n* = 176, 134 women, 25–59 years old)	Urdu literacy was positively associated with information seeking on web portals/through newsletter subscriptions OR 2.16 (95% CI 1.388–3.344), and communication on social networks OR 5.70 (95% CI 2.487–13.053), suggesting that eHealth services should be provided in minority languages rather than in English as a common language		X		
**Mixed-methods**					**HL**	**A**	**U**	**UA**
Smith-Miller, 2022 [46] USA	To explore gender-related factors related to T2DM self-management	SAHLSA to measure HL (0–50), ≤ 37 = low HL)	Latino immigrants recruited through flyers (*n* = 30, 19 women, 27–86 years old)*	Men received more self-management support than women. Family obligations and a lack of support impeded women’s self-management Mean (SD) scores: SAHLSA 37.9 (8.48) for men and 38.6 (8.69) for women	X			
**Experimental**					**HL**	**A**	**U**	**UA**
Chesla, 2013 [36] USA Single-group quasi-experimental (four-month delayed treatment) Intervention: Small groups receiving culturally adapted CST (six 2 h sessions, weeks 17–22)	To explore the efficacy of culturally adapted coping skills training (CST)	DQOL HbA1c.	Chinese immigrants recruited through the community (*n* = 145, 59% women, 36–83 years old)	The CST intervention improved personal, family, and disease management indicators of health Significant changes were observed in 7–10 outcomes from T3 to T4, suggesting that CST had immediate benefits of diabetes self-efficacy, diabetes knowledge, bicultural efficacy, family emotional support, family instrumental support, diabetes distress, and DQOL satisfaction. However, no significant improvements in glucose regulation (natural-log-transformed HbA1c) were observed		X		
Kim, 2020 [40] USA RCT (2012–2016) HL-focused T2DM self-management intervention: (1) weekly 2 h classes with self-management skill-building activities (six weeks), (2) monthly telephone counseling (behavioral coaching), and (3) daily monitoring of blood sugar at home	To examine mechanisms and the role of HL in diabetes management	REALM (0–66) and DM-REALM (0–82) to measure HL knowledge TOFHLA (0–7) and NVS (0–6) to measure functional HL skills Data collection: Baseline, 3, 6, 9, and 12 months	Korean immigrants with uncontrolled T2DM recruited from the community (*n* = 250) 209 completed the 12-month program (40.9% women; intervention: 105, control: 104), mean (SD) age 58.7 (8.4) years	HL-focused T2DM self-care management intervention positively influenced self-care (knowledge, self-efficacy, adherence, and glucose control) The mean (SE) HL levels were low at baseline: REALM 32.1 (1.5), DM-REALM 51.3 (1.7), TOFHLA functional HL 4.2 (0.2), NVS functional HL 1.7 (0.1). All measures showed an increase in HL during follow-up, except for TOFHLA at 6 and 12 months, suggesting that HL can be changed by relatively short interventions (< 3 months) The gaps in HL improvements expanded after adjusting for age, education, sex, financial stability, and years of U.S. residency. Self-efficacy and self-care skills mediated the relationships between HL and HbA1c and between HL and QoL. Education and acculturation were the most significant correlates (moderators) of HL	X			

* Smith-Miller et al. conducted multiple studies using the same dataset including 30 Latino Spanish-speaking immigrants (Mexicans: 25 and other Latin American countries: 5)

Findings related to HL and aspects of HL: access (A), understanding (U), and use/apply (UA)

CST: Coping Skills Training, CVD: Cardiovascular disease, DM-REALM: Diabetes Mellitus Rapid Estimate of Adult Literacy in Medicine, DQOL: Diabetes Quality of Life, HbA1c: Hemoglobin A1c, NVS: Newest Vital Sign, RCT: Randomized clinical trial, REALM: Rapid Estimate of Adult Literacy in Medicine, SAHLSA: Short Assessment of Health Literacy for Spanish-speaking Adults, SKILLD: Spoken Knowledge In Low Literacy in Diabetes, T2DM: Type 2 Diabetes Mellitus, and TOFHLA: Test of Functional Health Literacy in Adults

**Fig. 3:**
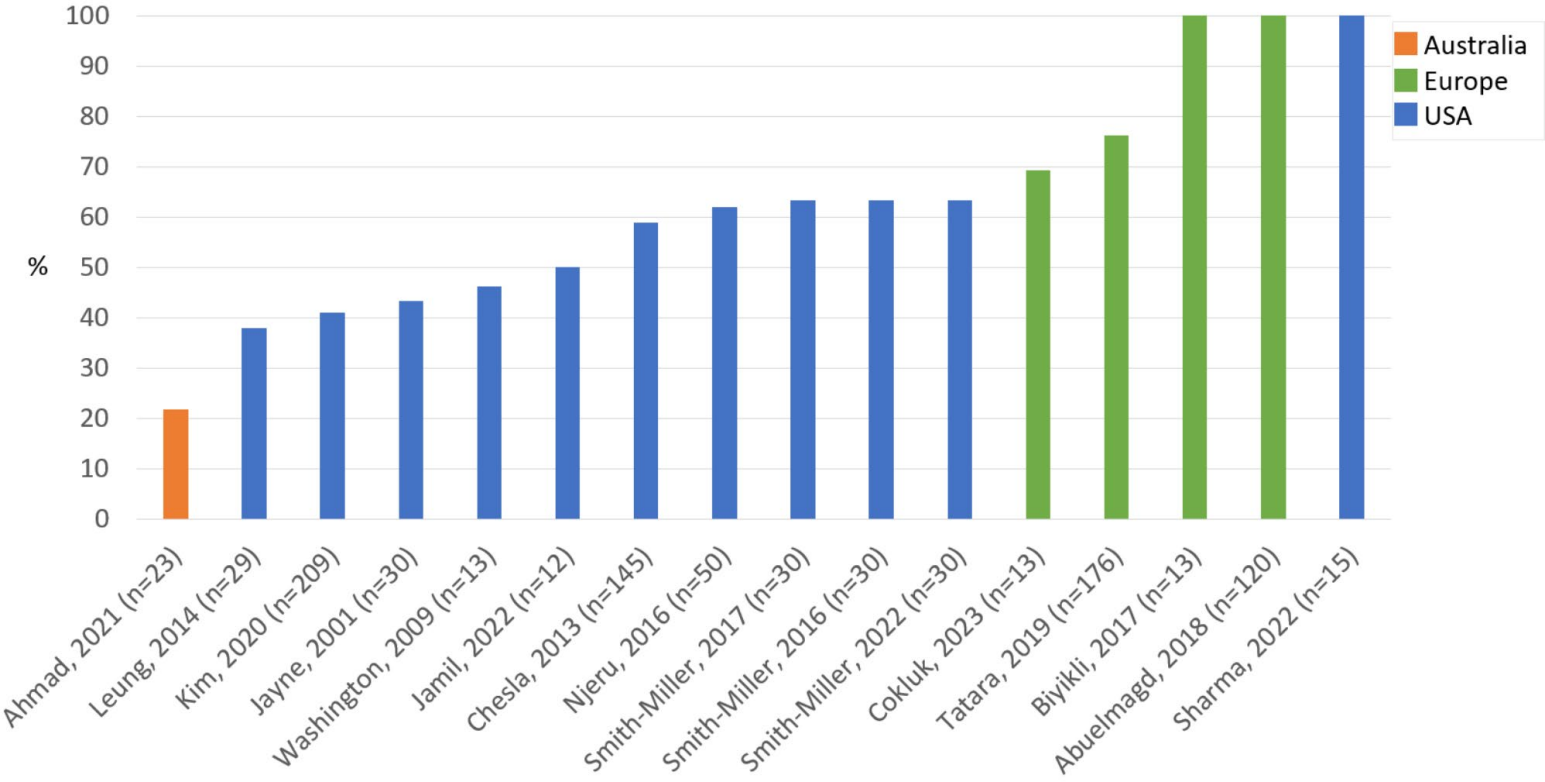
Women participants in the reviewed studies (%)

**Fig. 4:**
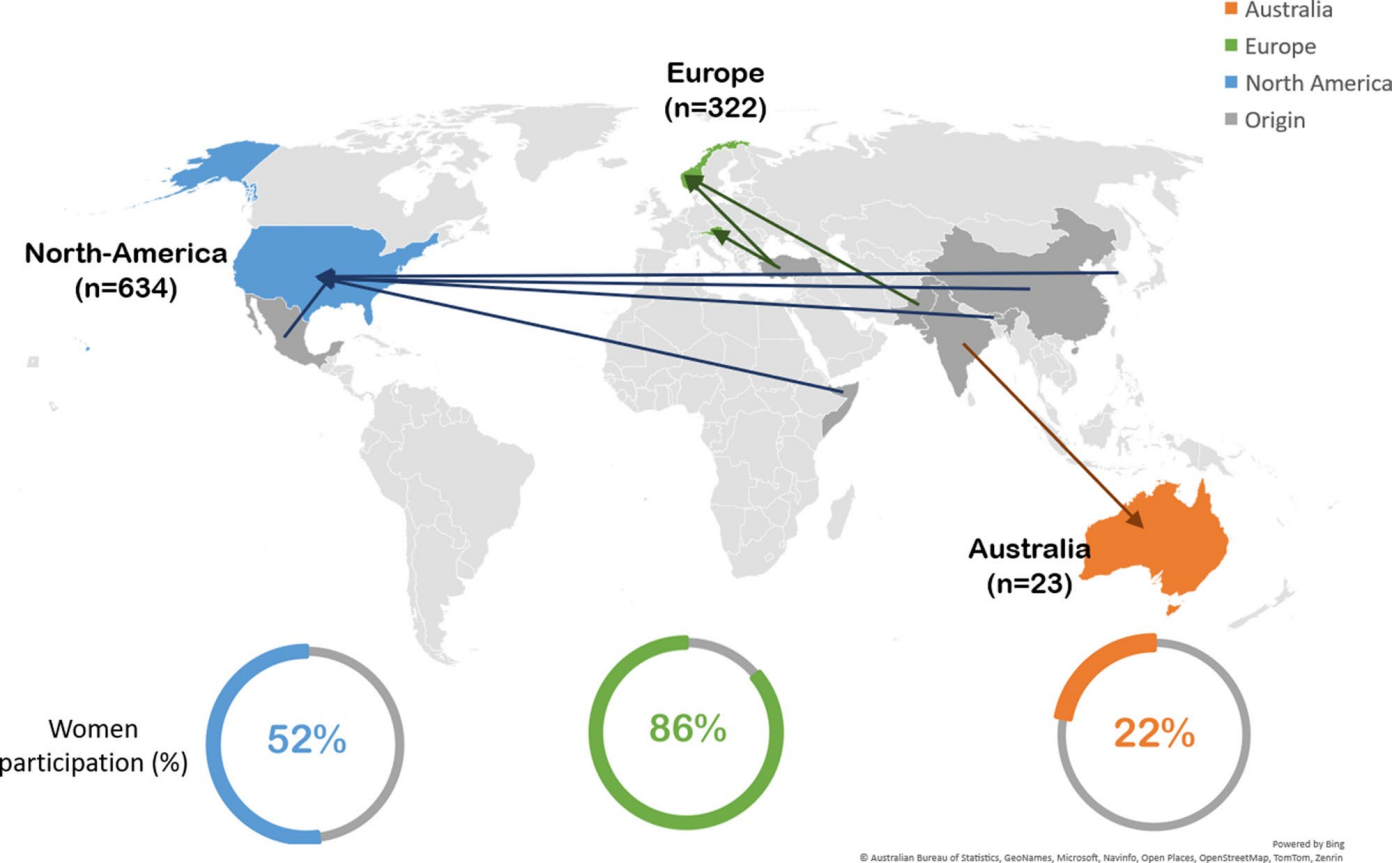
Immigrants’ host countries (blue, green, and orange), countries of origin (grey) and proportions of women participants in the various regions in the reviewed studies (*n* = 16)

Eleven of the studies were conducted in the USA [36, 38–46, 48], three in Norway [33, 37, 47], one in Austria [35], and one in Australia [34]. The number of participants varied between 12 and 209, of which 13 included both sexes (3–76% women), and three included only women [33, 35, 43] (Fig. 3). The participants, aged 20–86 years, originated from China [36, 39, 41, 48], India [34], Korea [40], Bhutan [43], Pakistan [33, 47], Somalia [42], Türkiye [35, 37], Latin America [44–46], and South Asia (Pakistan, India, and Bangladesh) [38] (Fig. 4).

### Results of individual studies related to research question

#### Health literacy

The included studies reported results concerning HL as a whole or aspects of HL (*access to*, *understanding of*, or *use/application* of health information) (Table 2). HL scores were reported in four papers, all conducted in the USA [40, 42, 44, 46], one RCT [40], two cross-sectional [42, 44], and one mixed-method study [46]. Overall, HL was low [40, 42] to moderate [44, 46], with no differences between the sexes [42, 46].

Njeru, 2016 [42] reported low HL levels among Somali immigrants and refugees (*n* = 50), where a low score was associated with higher age (+ 10 years), need for an interpreter, lower income, and diabetes-related complications with more retinopathy, neuropathy, and nephropathy. Only questions regarding signs of hypoglycemia, foot care, and eye examinations were answered correctly by at least 50% of the participants, while < 40% identified symptoms of hyperglycemia and < 20% regarding foot examination and normal glucose levels.

Smith-Miller 2016 [44] and 2022 [46] reported moderate HL (*n* = 30), with no differences between the sexes. Overall knowledge and healthy lifestyle behaviors were important components of their T2DM self-management.

In the RCT, Kim, 2020 [40] found low HL levels at baseline, and a significant increase in all HL measures during follow-up over the first three months. However, the improvements in functional HL were not significant at 6 or 12 months. Nevertheless, these findings suggest that HL can be changed by a relatively short (< 3 months) intervention. The gaps in HL improvements widened after adjusting for age, education, sex, financial stability, and years of residence in the host country. Self-efficacy and self-care skills mediated the relationship between HL and HbA1c and between HL and QoL, while education and acculturation were the most significant correlates of HL.

#### Aspects of health literacy

Twelve studies reported aspects of HL (*access to*, *understanding of*, or *use/application* of health information) (Table 2) [33–39, 41, 43, 45, 47, 48]: seven focused on access [33, 35–37, 41, 43, 47], nine focused on understanding [33–35, 38, 39, 41, 43, 45, 48], and three focused on use/applying of health information [34, 37, 41].

Ahmad, 2021 [34] reported that physicians’ advice motivated immigrants with T2DM (*n* = 23) to take their medication and that positive medicinal effects strengthened adherence. The key facilitator was understanding the importance of taking medicine as prescribed for improving outcomes. Health beliefs (fear of side effects and medication dependency, trust in Ayurvedic/alternative medicine), uncertainty (what one should do if one forgot to take the medication), and social stigma (delaying taking medicine due to discomfort of others knowing) negatively influenced adherence and the ability to use health information.

Biyikli Gültekin, 2017 [35] reported that limited knowledge affected the female participants’ approaches to the disease and identified challenges in accessing health information, including lack of language proficiency and low education and illiteracy levels, which limited their understanding. Due to language barriers, Turkish television/newspapers were the most important sources of health information (access). Moreover, diabetes education did not provide sufficient information on how to adapt when fasting (cultural adaption). Factors related to treatment discontinuation were physicians’ negative attitudes (bad physician-patient relation) and health beliefs (fear of side effects) that led to the use of herbal products.

Among Turkish immigrants, Cokluk, 2023 [37] health information access was primarily through physicians, with no diabetes education provided at diagnosis. Newspapers, social media, and Turkish TV were alternative information sources. Diet-related information application was challenging due to women’s family roles, such as preparing food for the whole family instead of diabetes-appropriate meals.

Jamil, 2022 [38] found that cultural and religious beliefs positively affected medication adherence among South Asian immigrants, who felt confident in managing their health decisions without physician involvement. They adjusted treatment based on their body’s responses, switched to familiar alternative medications, and sourced health information from personal networks, the internet, and self-monitoring. Good communication with healthcare providers enhances medication adherence. Clear blood sugar level goals motivated health-related changes.

Jayne, 2001 [39] reported a lack of understanding of the etiology of diabetes, and false health beliefs. The participants were reluctant to take insulin and some also used traditional Chinese medicine. Only half of the participants monitored their blood glucose levels.

Leung, 2014 [41] found that especially two factors seemed to influence all the aspects of HL (i.e., access, understand, and apply) among Chinese immigrants. There was limited diabetes information in the community undermining immigrants’ ability to access health information, and unawareness of self-care responsibilities made them passive in seeking health information and trying to understand the prescribed treatment regimens. Furthermore, age-related changes in older age limit their ability to obtain health information and communicate their needs.

Sharma, 2022 [43] found that patriarchal norms impacted Bhutanese women’s access to healthcare services and health information, and their autonomy in having a healthy lifestyle. They had poor HL and a limited understanding of diabetes due to illiteracy and language barriers, which complicate communication with healthcare providers and lead to reliance on family members who could speak the language to access healthcare information.

Smith-Miller, 2017 [45] reported uncertainty among participants regarding aspects of their medication regimens (e.g., insulin). Knowledge gaps were primarily related to the participants’ access to health information and understanding of medications and diet. Health beliefs influenced medication adherence leading participants to change medication dosages and diets.

Washington, 2009 [48] found that the immigrants’ ability to access diabetes-related information was limited by a lack of language proficiency and culturally sensitive health information. The insufficient understanding of necessary health information hinders effective diabetes self-care management.

Abuelmagd, 2018 [33] reported limited access to diabetes information among immigrants, where 30% had received no information about their T2DM, and 75% reported needing help to understand written medical information (mostly among those > 50 years without education). No differences were observed between participants with short and long (≤ 10 and > 10 years) residence in Norway. Almost 50% stated that they would stop taking medication due to religious beliefs.

In Norway, Tatara, 2019 [47] found that Pakistani immigrants with strong Urdu literacy frequently accessed eHealth services like web portals and health information subscriptions, while Norwegian proficiency correlated with using web/mobile apps for diabetes self-care. Despite half the participants reporting confidence in English, no association was found between English proficiency and eHealth access. This suggests that eHealth services should be provided in minority or local languages rather than in English as a common language.

Chesla, 2013 [36] reported beneficial effects on diabetes knowledge and self-efficacy after culturally adapted coping skills training (CST) in a group of Chinese Americans. They found that access to health information in the form of a two-hour T2DM-related video improved diabetes knowledge. Despite no significant improvement in glucose regulation.

### Synthesis of the results and implication for future research and practice

Overall, immigrants had low HL [40, 42, 44, 46] and limited health information [33, 37, 41], especially culturally adapted information (e.g., how to adapt when fasting) [35, 48], and low language proficiency [35, 43, 47, 48] seemed to affect their skills to access, understand, and apply health information. Some individuals were unaware of self-care responsibilities related to seeking health information [41]. Personal barriers such as age [40–42], gender [35, 37], sex, education, financial stability [42], and years of residence in the host country [40] can limit this access. Cultural norms, such as patriarchy [43] and respect for authority [41], can further restrict access to health information.

Language barriers limited in general, immigrants’ access to and understanding of health information [35, 41, 43, 48] and health information availability [33, 35, 37, 41, 43]. Access to health information, including diabetes education, often depends on physicians [35, 37]. For women, patriarchal norms limited this access, causing dependence on family members [43].

Most of the reviewed studies (*n* = 12) reported that immigrants struggled to understand health information due to cultural or religious health beliefs [33, 39, 45, 48], or limited knowledge of diabetes [33–35, 38, 39, 45] mostly due to insufficient diabetes information from health professionals [35, 37, 41].

Knowledge gaps primarily concerned medication and diet, with health beliefs and misconceptions influencing medication adherence and the use of alternative medicine [38, 39, 45]. However, also low knowledge of signs of complications was seen [42]. A lack of understanding of the importance of medication for improving diabetes outcomes could lead to non-adherence [34]. However, health professionals played a crucial role in motivating immigrants to adhere to treatment plans [34].

Moreover, a fear of side effects [34, 35], and medication dependence and concerns about social stigma (i.e., feeling uncomfortable with others knowing about their diagnosis) negatively influenced medication adherence and the ability to apply diet-related health information which was further affected by cultural norms related to having meals with family and friends that might be unsuitable for patients with T2DM [34, 37].

Effective communication with healthcare personnel can promote changes, such as improved adherence, and better health outcomes [38]. Culturally tailored educational videos are another resource that can enhance immigrants’ understanding of diabetes [36]. These videos could be further optimized by including self-management techniques, such as stress management, to increase participants’ knowledge and HL [36, 40].

**Fig. 5:**
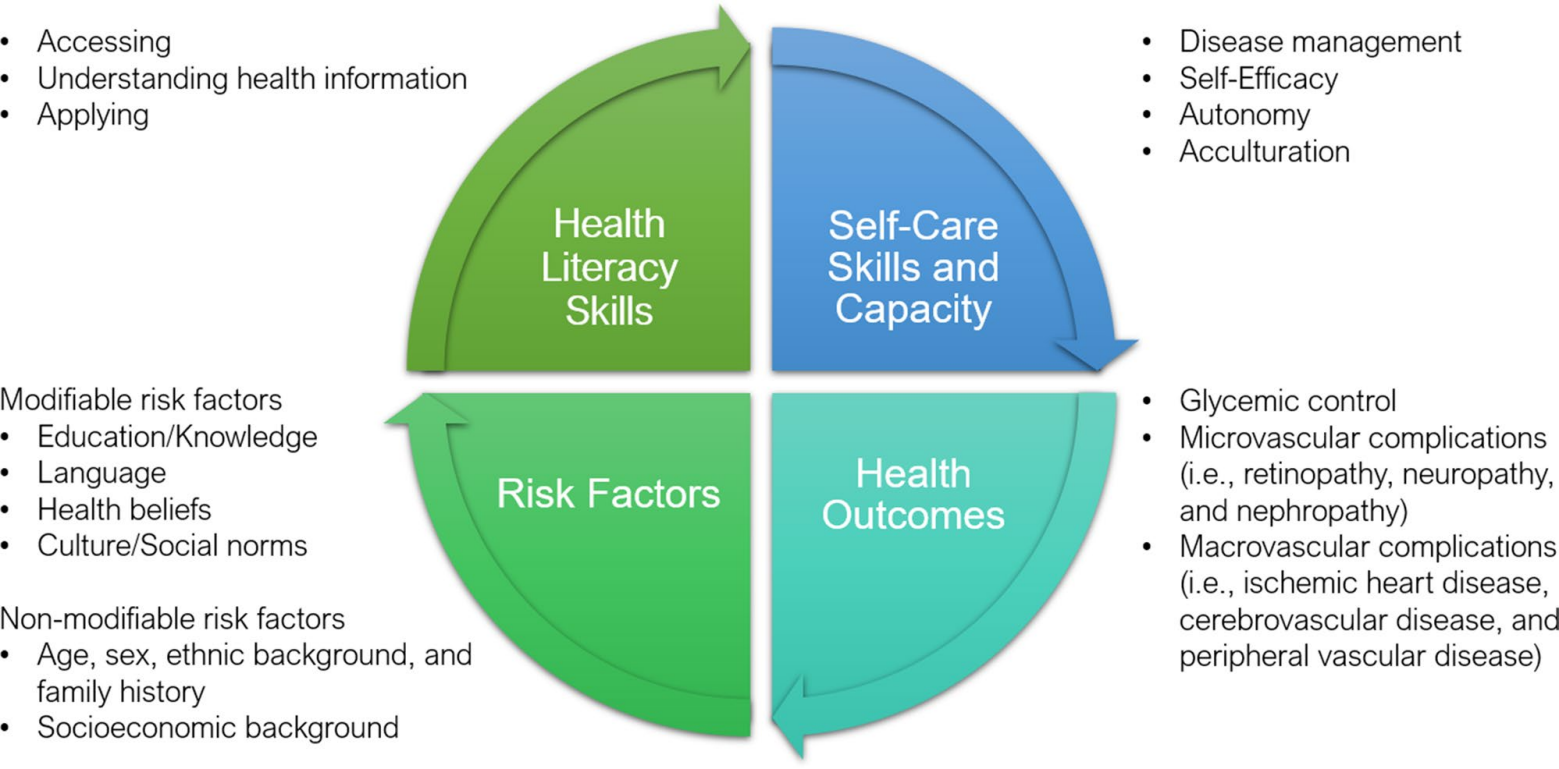
Diabetes health literacy model

According to Kim et al.’s theoretical framework, HL is a changeable construct that guides intervention development, focusing on self-care skills such as disease management and self-efficacy [40]. Operationalizing HL as a capacity can improve health outcomes such as glycemic control and QoL, through self-care skills [40]. To provide a theoretical roadmap we propose a framework considering risk factors for improving HL and self-care skills. Our proposed Diabetes Health Literacy Model (Fig. 5) incorporates factors influencing HL, that can be used to improve self-care skills and health outcomes among adult immigrants with T2DM.

In the proposed model, modifiable risk factors can be addressed through culturally adapted patient education that emphasizes skills necessary to comprehend and utilize health information. However, the usability of health information must be tailored to cater the non-modifiable risk factors. Initiatives such as diabetes courses can impact the modifiable risk factors, bolstering HL and subsequently enhancing individuals’ diabetes self-care skills. This can ultimately result in improved health outcomes, including glycemic control and reduced complications.

## Discussion

We found 16 articles on adult immigrants’ HL reporting of immigrants’ health beliefs, understanding, and self-management of T2DM. HL was generally low and was affected by limited health information from health professionals, language barriers, lack of culturally sensitive knowledge, cultural norms, and personal factors such as age, education, and income that influenced their health outcome.

Having specific knowledge about one’s chronic health condition is crucial. It influences HL and the ability to manage one’s health and impacts overall health outcomes [49]. Previously, ethnic differences in risk factors and total risk of CVD have been seen among immigrants in a Norwegian cross-sectional study [15]. CVD is highly prevalent in T2DM patients [50, 51]. Diabetes significantly increases adult mortality rates by 75%, largely due to CVD, and leads to numerous complications such as heart and renal disease, diabetic retinopathy, and cardiovascular autonomic neuropathy, all of which contribute to reduced QoL, disability, and early death [50]. CVD impacts approximately one-third of individuals with T2DM worldwide and accounts for about half of all deaths in this population, rendering it a leading cause of mortality among people with T2DM primarily due to coronary artery disease and stroke [52].

HL is closely linked to factors such as ethnicity, education, age, sex, gender, and disease severity, all of which play a role in managing T2DM as this review shows. This is in agreement with a recent review [28]. Some studies [41, 43] and a review by Nutbeam and Lloyd [16], suggest that a lower education level may result in reduced receptivity to health education, less utilization of disease prevention services, and difficulties in managing chronic illnesses. However, education alone may not guarantee that a person consistently uses health information in unfamiliar settings or situations that require specific knowledge and skills [16].

Cultural literacy promotes the ability to notice and use various beliefs, customs, and values as common interpretations of health information and related actions [53]. However, cultural factors, such as high regard for authority and collective approaches, can affect HL [41]. Regarding positive attitudes toward authority, Leung et al. highlighted Chinese T2DM patients’ respect for physicians’ opinions and advice. However, patients may choose to follow culturally conditioned advice and hide that they do not follow medical recommendations from physicians. Furthermore, women’s access to education and knowledge of their condition may be hindered by patriarchal norms [43].

Acculturation, language, food, friends, and identity are also pivotal for HL and T2DM self-management [36]. Low acculturation levels hinder effective T2DM management [48]. The level of acculturation affects adherence to treatment regimens including diet, physical activity, monitoring blood sugar, and medication which can be further complicated by cultural dietary norms [54].

Self-care skills such as adherence to treatment regimen, and lifestyle changes significantly impact T2DM management and outcomes [34, 38, 39, 45, 47]. A higher level of HL is associated with better adherence to medications, and diet, leading to improved glycemic control and outcomes of diabetes [55, 56].

Lifestyle changes after migration contribute to obesity and T2DM risk among immigrants [6], however, they often face barriers to lifestyle advice due to a lack of knowledge or gender norms [57]. Lifestyle choices are considered a part of self-care skills, however, they can be challenging to follow as shown in the reviewed studies [33, 48]. Immigrants may be aware of the impact of sugar consumption but appear ignorant of other dietary advice [48], and may not adhere to national physical activity guidelines for patients with T2DM (24).

Effective patient education strategies involve culturally sensitive approaches, language accessibility, and involving family members in the treatment [38, 41]. Healthcare providers should understand cultural norms, follow culturally sensitive HL guidelines, and communicate effectively [20, 58].

Health personnel, such as nurses, play a key role in improving patients’ HL by simplifying health information and adopting a HL approach that enhances patient care [28]. However, health personnel may sometimes struggle to identify patients at risk of low HL and may not always apply the recommended precautions when communicating with these patients [59]. Challenges in comprehending and utilizing health information can adversely affect the patient and escalate care costs. Professional responsibility in the context of HL involves awareness and understanding of the concept of HL and its impact on patient’s well-being, and the ability to adapt their communication and education strategy accordingly [60].

Social inequality, HL, and QoL are major public health indicators [16, 25, 61–63]. However, social inequality is often considered a contextual variable in studies exploring HL and QoL [64]. Nevertheless, HL may act as a mediating determinant of health, explaining the relationship between socioeconomic status and different health outcomes [16, 25], where individuals with higher education, superior jobs, and increased income usually have better access to health information and resources to utilize this information effectively [25]. Therefore, enhancing HL is crucial in tackling health inequalities [16]. Future initiatives should prioritize enhancing the quality and origin of health information and developing transferable skills like critical thinking, however, special attention should be given to population groups most impacted by low HL, aligning interventions with their specific needs [16].

### Limitations

Only articles published in English were included in this review. Despite a comprehensive search in multiple databases, relevant articles published in other languages or using keywords not included in our search strategy may have been missed. To mitigate this limitation, an academic librarian (MWG) with extensive experience assisted in the search process to ensure its quality. Another limitation is the absence of a registered protocol for this project. Only a one-page plan outlining the search strategy was used. However, the project adhered to the methodological framework developed by Arksey and O’Malley (2005) [29], and Levac (2010) [30], and the reporting followed the PRISMA-ScR guidelines [31]. Moreover, to enhance reliability, both authors were involved in the review process independently screening all papers, and having weekly sessions to ensure consistency in screening decisions.

## Conclusions

This is the first scoping review focusing on HL and self-care behaviors among adult immigrants with T2DM. Few studies were found on this topic, and additional research is needed to enhance HL among immigrants. The included studies report generally low HL, impacted by limited health information, language barriers, and lack of culturally sensitive knowledge, where cultural norms and personal factors further affected the participants’ health outcomes. The study suggests that health professionals should have communication skills and knowledge of HL in their curricula to identify and support patients with low health literacy.

## Data Availability

No datasets were generated or analysed during the current study.
